# Characterization of a Novel Dermal Fibrosis Model Induced by Areca Nut Extract that Mimics Oral Submucous Fibrosis

**DOI:** 10.1371/journal.pone.0166454

**Published:** 2016-11-16

**Authors:** Min-Hsuan Chiang, Ping-Ho Chen, Yuk-Kwan Chen, Chia-Hsin Chen, Mei-Ling Ho, Yan-Hsiung Wang

**Affiliations:** 1 School of Dentistry, College of Dental Medicine, Kaohsiung Medical University, Kaohsiung, Taiwan, Republic of China; 2 Orthopaedic Research Center, College of Medicine, Kaohsiung Medical University, Kaohsiung, Taiwan, Republic of China; 3 Institute of Biomedical Sciences, National Sun Yat-sen University, Kaohsiung, Taiwan, Republic of China; 4 Cancer Center, Kaohsiung Medical University Hospital, Kaohsiung Medical University, Kaohsiung, Taiwan, Republic of China; 5 Center for Infectious Disease and Cancer Research (CICAR), Kaohsiung Medical University, Kaohsiung, Taiwan, Republic of China; 6 Oral & Maxillofacial Imaging Center, Kaohsiung Medical University, Kaohsiung, Taiwan, Republic of China; 7 Division of Oral Pathology & Maxillofacial Radiology, Department of Dentistry, Kaohsiung Medical University Hospital, Kaohsiung, Taiwan, Republic of China; 8 Department of Physical Medicine and Rehabilitation, Kaohsiung Municipal Ta-Tung Hospital, Kaohsiung Medical University, Kaohsiung, Taiwan, Republic of China; 9 Department of Physical Medicine and Rehabilitation, Kaohsiung Medical University Hospital, Kaohsiung Medical University, Kaohsiung, Taiwan, Republic of China; 10 Department of Physiology, College of Medicine, Kaohsiung Medical University, Kaohsiung, Taiwan, Republic of China; Taipei Medical University, TAIWAN

## Abstract

Oral submucous fibrosis (OSF) is an oral potentially malignant disorder and areca quid chewing is the main etiological factor. However, the molecular mechanism underlying OSF remains unclear, partly due to the lack of an appropriate animal model. The present study aimed to establish and characterize an animal model of areca nut extract (ANE)-induced skin fibrosis that mimics OSF. Mice were divided into 4 groups: the control group; the bleomycin group; and the ANE10 and ANE20 groups, which received 10mg/ml and 20mg/ml subcutaneous (SC) injection of ANE, respectively. Skin fibrosis was evaluated by histological analyses. Additionally, the expression levels of the fibrotic marker genes were determined by immunohistochemical staining and immunoblotting. ANE administration significantly increased dermal thickness and collagen deposition compared with the control group. Moreover, ANE induced the expression of the fibrotic marker genes alpha smooth muscle actin (α-SMA) and connective tissue growth factor (CTGF) in the skin lesions. The SC injection of ANE successfully induced skin fibrosis, exhibiting characteristics similar to those of OSF. This model may facilitate future studies of the mechanism underlying OSF.

## Introduction

Oral submucous fibrosis (OSF) is an oral potentially malignant disorder [1]with a high risk of malignant transformation [1]. Several studies have indicated that 7–30% of OSF patients may experience transformation into oral squamous cell carcinoma (OSCC) [2–4]. Similar to fibroses in other tissues, excessive depositions of extracellular matrix (ECM) components are observed in OSF lesions. However, the molecular mechanism of areca nut chewing-induced OSF remains unclear, and the majority of the related information was collected based on clinical and in vitro studies. The possible etiological factors that have been indicated in the development of OSF include vitamin deficiencies, tobacco use, areca nut chewing, and genetic disorders. A number of lines of epidemiological evidence and clinical studies strongly indicate that areca quid chewing is the main etiological factor in the development of OSF [5–12].

However, many in vitro studies have indicated that ANE increases collagen formation via the transdifferentiation of myofibroblasts that express the intracellular marker protein alpha smooth muscle actin (α-SMA) and further decrease collagen degradation via the modulation of matrix metalloproteinase (MMP) activity [13–15].Furthermore, ANE may induce fibrosis through transforming growth factor-beta (TGF-β), connective tissue growth factor (CTGF), interleukin 6 (IL-6), and prostaglandin E2 (PGE2) signaling factors in different cell lines. CTGF, also known as CCN2, is a multifunctional heparin-binding glycoprotein that is normally expressed at low levels but is increased in fibrotic tissues [16]. CTGF may enhance fibroblast proliferation, migration, adhesion, and ECM production. CTGF may also play roles in OSF development [17–21].

To our knowledge, only one ANE-induced OSF animal model has been reported based on a literature review. In 2007, Sumeth Perera and coworkers demonstrated that after 300 to 600 days of ANE application twice daily to the buccal mucosa of mice, OSF development could be observed [22]. However, the establishment in one month of bleomycin-induced dermal fibrosis in mice via SC injection has been well established [23]. Here, we investigated whether SC injection of ANE could induce dermal fibrosis mimicking the pathologic development of OSF.

## Materials and Methods

### Areca nut extract

The areca nut extract (ANE) was purchased from the Haw Yaun Vacuum- Biochemical- Technology CO., LTD. (Taoyuan, Taiwan). The lyophilized ANE was dissolved in PBS, passed through 0.22-μm filters and then stored at -80°C until use.

### Animal experiments

The animal experiments were approved by the Animal Care and Use Committee of Kaohsiung Medical University (Affidavit of approval of animal use protocol number: 103149). Ninety-six specific pathogen-free 6-week-old male BALB/C mice (20 g) were purchased from LASCO (Bio Lasco Taiwan Co. Ltd., Taipei, Taiwan) and housed under standard laboratory conditions with food and water provided libitum. Equal-volume (100μl) drug treatments were injected subcutaneously into areas of approximately 1 cm^2^ on the shaved backs of the mice every other day until the mice were killed.

The mice were divided into the following four groups: PBS (control group, PBS injection, n = 24), BLM (0.5 mg/ml bleomycin injection, n = 24), ANE10 (10 mg/ml ANE injection, n = 24), and ANE20 (20 mg/ml ANE injection, n = 24). Six mice from each group were killed via CO_2_inhalation after the treatments had been administered for 3 days, 7days, 14 days, and 30 days. The skin tissues were harvested, and each tissue sample was divided into 2 parts for histology and immunoblot analysis.

### Histomorphological analysis

The skin tissues were fixed in 10% formalin. Following fixation, the skin tissues were dehydrated and embedded in paraffin wax according to standard procedures. Serial 5-μm sections were obtained and routinely stained with hematoxylin/eosin (Sigma-Aldrich) and Masson’s trichrome. The dermal thickness was defined as the mean width between the epidermal–dermal junction and the dermal–SC fat junction. The collagen tissues in the skin sections were stained blue with Masson’s trichrome stain. The dermal thickness and the ratio of collagen tissue in the dermal section were analyzed using Image-Pro Plus software version 5.0.

### Immunohistochemistry

The dermal sections were deparaffinized and dehydrated, and the endogenous peroxidase activity was then quenched with freshly prepared 1% H_2_O_2_. The sections were treated with target retrieval solution (Sigma-Aldrich), incubated with serum-free blocking agent (DAKO), and subsequently incubated with anti-CTGF (Santa Cruz) and anti-α-SMA (Santa Cruz) primary antibodies overnight at 4°C.

Isotype-matched irrelevant IgG (Thermo) antibodies at the same concentration were substituted for the primary antibodies in the negative controls. The bound antibodies were visualized using horseradish peroxidase-conjugated secondary antibodies (Dako). After counterstaining with hematoxylin, the sections were mounted and observed with a microscope. The relative densities of immunostaining were measured with Image-Pro Plus software version 5.0.

### Immunoblotting

The proteins were extracted from the skin tissue using RIPA lysis buffer (Sigma-Aldrich). Concentrations equivalent to 40 μg of protein were determined using a BCA protein assay kit (Thermo). The proteins were analyzed on a 12% SDS-PAGE gel and transferred to a PVDF membrane with the Bio-Rad system. The membrane was blocked in phosphate-buffered saline-0.5% Tween 20 (PBS-T) containing 5% non-fat milk and subsequently incubated with the primary antibodies, i.e., α-SMA (Cell Signaling) and CTGF (Thermo), overnight at 4°C. The membrane was also probed with β-actin (Merck) as the loading control. The bound antibodies were visualized using horseradish peroxidase-conjugated secondary antibodies (Santa Cruz) and ECL reagent (Thermo). The bands were compared to molecular weight standards (Thermo) to confirm the appropriate sizes of the proteins. All membranes were detected using a chemiluminescence detection system (UVP).

### Statistical analysis

SPSS version 17.0 was used for the statistical analyses. The results are expressed as the mean± standard deviation. Statistically significant differences were determined by analysis of variance (ANOVA) followed by Tukey’s post-hoc tests for multiple comparisons. A p-value less than 0.05 was considered statistically significant.

## Results

### SC injection of ANE increased dermal thickness

**Fig 1:**
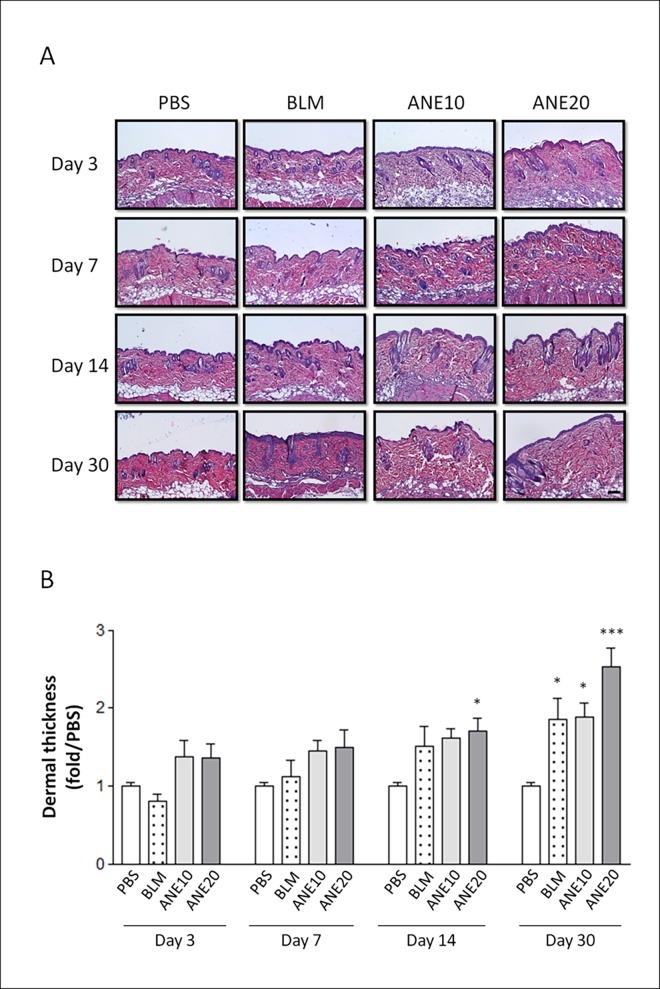
Histopathological evaluation of SC-injected ANE-induced changes in dermal thickness. (A.) The skin injection sites were examined by H&E staining on days 3, 7, 14, and 30. The sections were viewed under a microscope at 100x magnification. Scale bars indicate 100 μm. (B). Quantification of the average dermal thickness of the tissue using IMAGE-Pro software. Each treatment group included at least 6 mice. The results are shown as the mean± SD. The levels of statistical significance are as follows: *P<0.05, **P<0.01 and ***P<0.001 relative to the control group.

To investigate whether SC injection of ANE induced dermal fibrosis, mice received different dosages of ANE, and the morphologic changes were analyzed at different time points. The mice that received the BLM treatment exhibited notable dermal thickening and fat layer shrinkage, whereas the phosphate buffered saline (PBS) injections did not cause any morphological changes (Fig 1A). Interestingly, within one week, the ANE treatment groups, i.e., ANE10 (1.45±0.28) and ANE20 (1.5±0.45), exhibited obviously thicker skins compared with the PBS group (Fig 1A). Measurements of the dermal thickness revealed that the ANE and BLM treatment groups exhibited time-dependent increases in dermal thickness. Compared with the PBS group, the dermal thickness in the ANE20 (1.7±0.3, P<0.05) was significantly greater on day 14. The dermal thickness of the BLM (1.86±0.52, P<0.05), ANE10 (1.88±0.36, P<0.05), or ANE20 (2.53±0.48, P<0.001) group was also significantly greater than that of the control group on day 30. Moreover, the ANE20 group exhibited greater dermal thickness than the ANE10 group.

### SC injection of ANE induced collagen deposition

**Fig 2:**
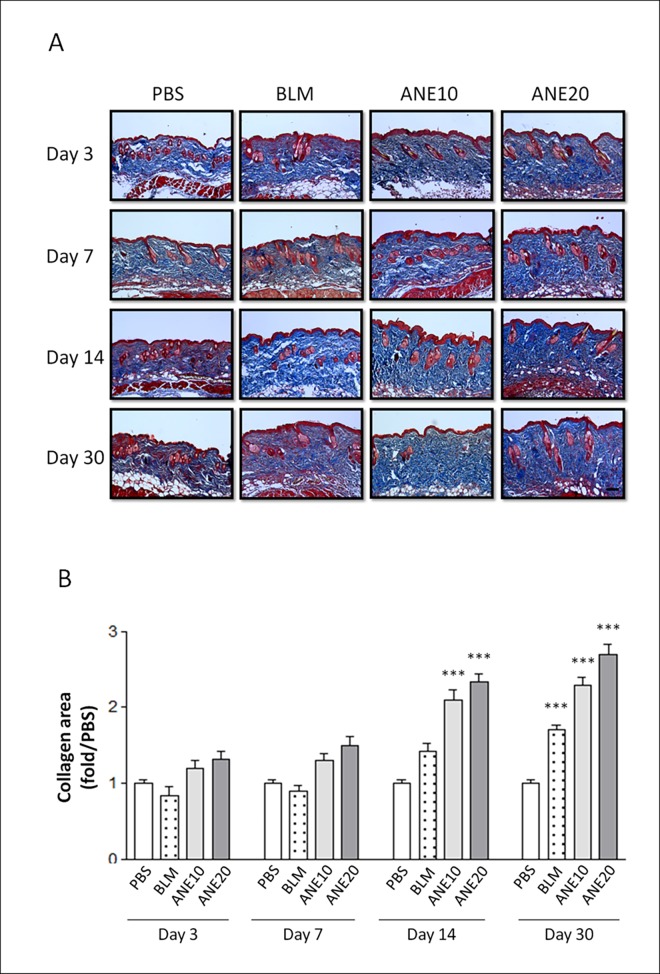
Histopathologic evaluation of the SC-injected ANE-induced changes in dermal collagen synthesis. (A).The skin injection sites were examined on days 3, 7, 14, and 30by Masson’s trichrome staining. The sections were viewed under a microscope at 100x magnification. Scale bars indicate 100 μm. (B). the intensity of the blue color representing the collagen density was measured using IMAGE-Pro software. Each treatment group included at least 6 mice. The results are shown as the mean± SD. The levels of statistical significance are as follows: *P<0.05, **P<0.01 and ***P<0.001 relative to the control group.

Collagen deposition is a major characteristic of tissue fibrosis. Thus, we further analyzed collagen deposition by Masson’s trichrome staining. The collagen fibers were stained blue (Fig 2A). Measurement of the collagen fiber area was normalized to that of the control group, and the results showed that the ANE treatment groups exhibited time-dependent increases in collagen deposition. Furthermore, both the ANE10 and ANE20 groups exhibited significantly greater collagen staining than the control group on days 14 [ANE10 (2.1±0.26, P<0.001) and ANE20 (2.33±0.21, P<0.001)], and 30 [(ANE10 (2.29±0.2, P<0.001) and ANE20 (2.7±0.25, P<0.001)]. The BLM-treated group also exhibited significantly stronger collagen staining than the control group on day 30 (1.7±0.12, P<0.001) (Fig 2B). The ANE20 group exhibited a greater than 2.7-fold increase in skin collagen content, and the BLM group exhibited an approximately 1.7-fold increase in skin collagen content on day 30.

### SC injection of ANE increased the expression of fibrotic growth factors

**Fig 3:**
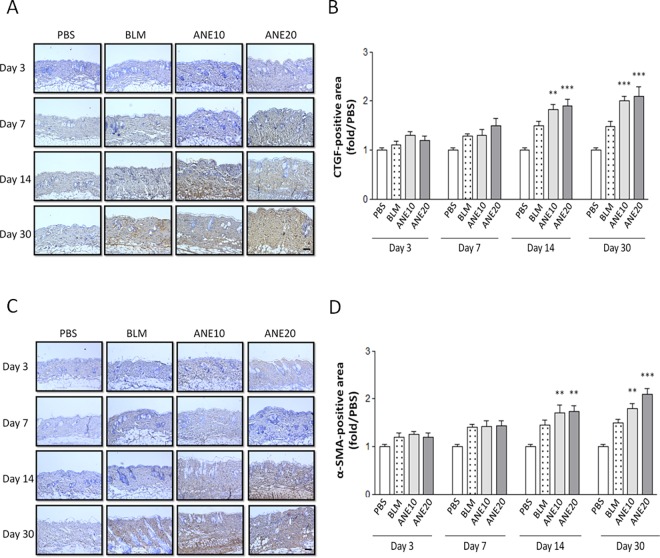
SC injection of ANE induces fibrotic gene immunoreactive-positive tissue expression. (A). CTGF immunoreactivities were noted at the ANE injection sites on days 3, 7, 14, and 30. (B). Measurement of the CTGF immunoreactive-positive tissues were obtained from 6 random areas, and the number of brown-stained tissue was determined using IMAGE-Pro software. (C). α-SMA immunereactivities were noted at the ANE injection sites on days 3, 7, 14, and 30. (D). Measurement of α-SMA immunoreactive-positive tissues were obtained from 6 random areas, and the number of brown-stained tissues were assessed using IMAGE-Pro software. The sections were viewed under a microscope at 100x magnification. Scale bars indicate 100 μm. Each treatment group included at least 6 mice. The results are shown as the mean± SD. The levels of statistical significance are as follows: *P<0.05, **P<0.01 and ***P<0.001 relative to the control group.

**Fig 4:**
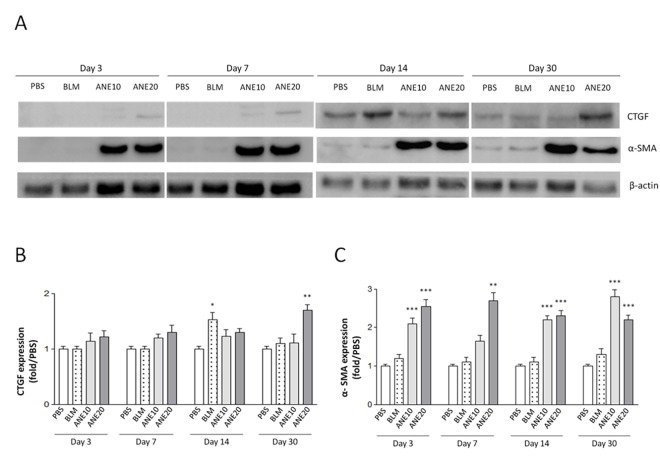
SC injection of ANE induces fibrotic protein expression. At 3, 7, 14, and 30 days after ANE injection, the expression levels of CTGF and α-SMA (A) protein were examined by immunoblotting. Quantification of CTGF (B) and α-SMA (C) protein expression using IMAGE-Pro software. Each treatment group included at least 6 mice. The results are shown as the mean± SD. The levels of statistical significance are as follows: *P<0.05, **P<0.01 and ***P<0.001 relative to the control group.

Next, we used immunohistochemical and immunoblotting methods to examine whether ANE and BLM induced the expression of fibrotic growth factors. The PBS injections did not affect the expression of CTGF (Fig 3A) or α-SMA (Fig 3C), and a weak staining signal was observed. Time-dependent increases in CTGF expression were observed following the BLM and ANE treatments (Fig 3A). Next, the stained signals were further quantified. Compared with the PBS group, ANE-induced CTGF expression was significantly increased in the ANE10 and ANE20 groups on day 14 [ANE10 (1.82±0.23, P<0.01) and ANE20 (1.9±0.28, P<0.001)] and day 30 [ANE10 (2.04±0.2, P<0.001) and ANE20 (2.1±0.38, P<0.001)] (Fig 3B). Consistently, similar α-SMA expression patterns were observed, day 14 [ANE10 (1.7±0.35, P<0.01) and ANE20 (1.74±0.24, P<0.001)] and day 30 [ANE10 (1.8±0.19, P<0.001) and ANE20 (2.2±0.22, P<0.001)], as illustrated in Fig 3C&3D. On the other hand from the immunoblotting results, only the ANE20 group exhibited a significant increase in CTGF expression on day 30 (1.7±0.22, P<0.01) (Fig 4A&4B). Additionally, the ANE20 group exhibited significantly stronger α-SMA signals as compared with the control group on day 3 (2.54±0.38, P<0.001), day 7 (2.7±0.4, P<0.001), day 14 (2.3±0.29, P<0.001) and day 30 (2.2±0.25, P<0.001) (Fig 4A&4C). BLM increased the expression of CTGF and α-SMA, but these increases did not reach statistical significance (Fig 4).

## Discussion

The areca nut contains alkaloids, flavonoids, and copper, all of which may interfere with the homeostasis of the ECM and induce OSF. Three main alkaloids, i.e., arecoline, arecidine, and quvacine, are known to stimulate collagen synthesis in fibroblasts and lead to OSF [24]. Flavonoids, tannins and catechins may inhibit MMP activities and, consequently, result in reduced ECM degradation [24,25]. In the present study, we found that SC injection of ANE induced dermal fibrosis in mice. Further studies are required to clarify the ANE component that functions as the key inducer of fibrosis.

Animal models of scleroderma induced by repeated SC injections of BLM have been established in various mouse strains. These mice exhibit dermal sclerosis comprising thickened and homogeneous collagen bundles [26–28]. Additionally, sclerotic changes have not been observed in skin sites distant from the injection site [23]. In the present study, BLM treatment was used as a positive control and elicited greater thickening and more condensed collagen deposition than PBS treatment. These are the first data to show that ANE can induce dermal fibrosis. Interestingly, the ANE treatments not only induced dermal fibrosis but also generated stronger fibrotic effects within one month compared with the BLM treatment. Moreover, the ANE-induced fibrosis was produced in a dose-dependent manner (Figs 1 and 2). Finally, BLM and ANE treatments increased the levels of fibrotic proteins (Fig 4).

CTGF protein levels are increased in most fibrotic diseases, and many studies have demonstrated that CTGF plays an important role in the pathological processes of fibrosis [29]. Moreover, activated fibroblasts and myofibroblasts, which are characterized by the expression of α-SMA, can be stimulated and observed in fibrotic tissue [30]. Several studies have indicated that the expression of α-SMA may be regulated by different signaling pathways, e.g., the TGF-β, CTGF and cAMP/PKA pathways [31]. The immunoblot analyses revealed that the expression levels of both CTGF and α-SMA were increased following ANE treatment (Fig 4). These results further confirmed that ANE could induce fibrotic marker gene expression and induce dermal fibrosis. However, CTGF and α-SMA exhibited different expression patterns, indicating that different factors may be involved the regulation of these two fibrotic genes.

The purpose of this study was to develop a stable ANE-induced animal model of fibrosis within a time period that is appropriate for preclinical testing. The only OSF model that has been reported is that described by Sumeth Perera and coworkers in 2007. In that model, the buccal mucosa was treated twice daily 6 days per week with a topical application of aqueous ANE for 300–600 days. The authors of the study demonstrated that after only 600 days of treatment, the fibrotic scores for cellularity, inflammation and muscle atrophy were significantly different from those of the control group [22]. However, this model is time-consuming and requires almost the entire life span of a mouse to induce OSF. Many confounding factors (particularly aging) may affect OSF development in this model. Moreover, due to the long generation time and uncertainty regarding therapeutic interventions for OSF, this model is difficult to apply for the validation of therapeutic OSF treatments. In our study, dermal fibrosis was induced by ANE injections within 4 weeks. In a pilot study, we also found that ANE injections into the buccal tissue induced fibrosis within 4 weeks (data not shown), and similar pathological features of OSF were observed. However, there were some potential limitations to this study. The tissues are phenotypically difference between the oral mucosa and skin. Thus, the mechanism of the dermal fibrosis model may different from OSF [32–34]. Further study is required to understand the effects of epithelium-stroma interaction and physical irritation in our animal model.

Based on our results, we have established a stable and rapidly generated ANE-induced model of fibrosis. Further investigations are underway to identify the mechanisms underlying ANE-induced fibrosis in the skin and buccal tissue. Areca nut chewing-induced OSF has been associated with a high risk of malignant transformation. Therefore, we are also studying the potential for cancer formation via long-term follow-ups of the ANE-induced model mice. We hope that this model will not only improve our understanding of ANE-induced fibrosis but also provide a prototype for the development of therapeutic interventions for OSF. Our model may also be suitable for studies of the relationship between ANE-induced fibrosis and the development of cancer.

## Abbreviations

OSF: Oral submucous fibrosis
ANE: Areca nut extract
SC: Subcutaneous
BLM: Bleomycin
PBS: Phosphate buffered saline
OSCC: Oral squamous cell carcinoma
ECM: Extracellular matrix
α-SMA: Alpha smooth muscle actin
MMP: Matrix metalloproteinase
TGF-β: Transforming growth factor-beta
CTGF: CCN2, Connective tissue growth factor
IL-6: Interleukin 6
PGE2: Prostaglandin E2
